# Osteoprotegerin and Inflammation in Incident Peritoneal Dialysis Patients

**DOI:** 10.3390/jcm13082345

**Published:** 2024-04-18

**Authors:** Michał Małecki, Patrycja Okulewicz, Marcin Lisak, Krzysztof Safranow, Leszek Domański, Kazimierz Ciechanowski, Edyta Gołembiewska

**Affiliations:** 1 Department of Nephrology, Transplantology and Internal Medicine, Pomeranian Medical University, Al. Powstańców Wlkp. 72, 70-111 Szczecin, Poland; 2 Department of Biochemistry and Medical Chemistry, Pomeranian Medical University, Al. Powstańców Wlkp. 72, 70-111 Szczecin, Poland

**Keywords:** chronic kidney disease, osteoprotegerin, peritoneal dialysis, inflammation

## Abstract

**Objectives**: Osteoprotegerin (OPG) is a member of the tumor necrosis factor receptor family involved in processes in many inflammatory states. OPG concentration is enhanced in the majority of chronic kidney disease (CKD) patients and those undergoing renal replacement therapy. The aim of the study was to assess the relation of OPG and chronic inflammation in peritoneal dialysis (PD) patients and to evaluate whether OPG concentrations in plasma and dialysate were related to plasma and dialysate levels of proinflammatory mediators (interleukin 6 (IL-6), high-sensitivity C-reactive protein (hsCRP), interleukin 33 (IL-33) and interleukin 1 receptor-like 1IL-1RL1 (IL-1RL1, sST2)). **Methods**: The study included 37 patients of the Peritoneal Dialysis Center, Department of Nephrology, Transplantology and Internal Medicine, Szczecin, Poland, 4–6 weeks after the onset of peritoneal dialysis therapy. During a peritoneal equilibration test, plasma (at 2 h) and dialysate (at 4 h) OPG, IL-33, 1IL-1RL1 (sST2), IL-6 and hsCRP concentrations were determined. **Results**: Plasma concentration of OPG did not correlate with dialysate OPG level (*Rs* = 0.04, *p* = 0.8). There was a strong positive correlation between plasma OPG concentrations and plasma IL-1RL1 (sST2) (*Rs* = 0.41; *p* = 0.01), plasma IL-6 (*Rs* = 0.38; *p* = 0.01) and plasma hsCRP (*Rs* = 0.35; *p* = 0.02). Dialysate OPG concentrations were positively associated with dialysate IL-1RL1 (sST2) (*Rs* = 0.37; *p* = 0.02) and dialysate IL-6 levels (*Rs* = 0.44; *p* = 0.005). Multivariate analysis showed that higher IL-1RL1 (sST2) (*ß* = +0.38, *p* = 0.006), higher plasma hsCRP (*ß* = +0.32, *p* = 0.02) and older age (*ß* = +0.35, *p* = 0.01) were independent determinants of higher plasma OPG concentration and that higher concentrations of dialysate IL-6 (*ß* = +0.37, *p* = 0.02) were independent determinants of higher dialysate OPG concentration. **Conclusions**: Both plasma and dialysate OPG levels are associated with the severity of systemic and local inflammation illustrated by the plasma and dialysate concentrations of IL-1RL1 (sST2), hsCRP and IL-6, suggesting that OPG might have a pivotal role in explaining the milieu of systemic and intraperitoneal inflammation.

## 1. Introduction

Chronic kidney disease (CKD) is regarded as a leading public health issue reported to affect 10.6% of the global population [1]. Kidney Disease Improving Global Outcomes (KDIGO) 2012 defines CKD as “abnormalities of kidney structure or function, present for >3 months, with implications for health” [2]. Chronic inflammation is recognized as a main cause of complications in CKD patients, specifically cardio-vascular events. There is increasing evidence associating the incidence and severity of chronic inflammation with disease progression. This correlation is widely observed among patients treated with renal replacement therapies [3]. Many factors contribute to permanent activation of inflammation, including accumulation of uremic toxins, oxidative stress, hypoxia, overhydration and sodium overload, genetic predisposition, gut dysbiosis and conducting dialysis procedures due to central venous catheters and membrane bioincompatibility [4].

According to different studies, inflammation, illustrated by the number of plasma biomarkers, increases progressively with renal function deterioration. Elevated concentrations of C-reactive protein (CRP) and interleukin-6 (IL-6) are strictly correlated to higher comorbidity, poor quality of life and high overall mortality in this group of patients [5]. Moreover, interleukin-33 (IL-33) and interleukin 1 receptor-like 1 (IL-1RL1; sST2) are linked to endothelial dysfunction and therefore higher cardiovascular risk [6]. Endothelial cells exposed to cytokines promote expression of cell-surface adhesion molecules and intensify the development of atherosclerotic lesions in the arteries [6].

In peritoneal dialysis (PD), a specific, rich-in-,glucose, solution is introduced into the peritoneum, allowing uremic toxins and excess fluid to diffuse across the peritoneal membrane acting as a filter [7]. Bioincompatible fluids contribute to the activation of local inflammatory processes (mainly due to the low pH, hyperosmolality and the presence of glucose degradation products) and exert detrimental effects on the peritoneal membrane, leading to the damage of its structure and gradually dialysis method failure [7,8,9,10].

Osteoprotegerin (OPG), first discovered in 1997, is a member of the tumor necrosis factor receptor family secreted by B cells, mediating powerful effects on bone [11]. OPG has been shown to act as a decoy receptor for RANKL (RANK ligand) and consequently disrupt the differentiation of osteoclast precursor cells into mature osteoclasts.

Numerous studies suggest that OPG promotes inflammation; however, the exact role which OPG plays in the Immuno-Skeletal Interface (ISI) is far from fully understood. In several inflammatory states, B cells and T cells disrupt the ISI and promote bone resorption through regulation of RANKL, OPG and inflammatory cytokines [12,13]. The impact that OPG exerts on atherosclerosis, the process strictly connected with chronic inflammation, is an intensely studied topic since the OPG/RANKL ratio was found to correlate with endothelial dysfunction progression [14]. Osteoprotegerin may act as a biomarker of vascular calcification and cardiovascular risk in patients on PD, as it was shown to predict carotid artery intima-media thickness [15] and is positively correlated with aortic pulse wave velocity [16]. The concentration of OPG generally increases in inflammatory conditions. Studies in in vivo models revealed that OPG secretion is stimulated by key proinflammatory mediators, such as interleukin-1 (IL-1) and IL-6 [13,14]. The levels of OPG are enhanced in the vast majority of CKD patients, increasing with a decline in eGFR in a proportional manner [17,18,19]. Even though this correlation is specifically vivid in CKD stages 3 to 5, a significant increase in OPG concentration was observed in CKD stage 2 as well [18]. In patients with end-stage renal disease (ESRD) undergoing renal replacement therapy, OPG concentrations are elevated, both in the group treated with peritoneal dialysis and in those performing hemodialysis [15].

In this study, we aimed to assess whether the OPG concentrations in plasma and dialysate are related to plasma and dialysate levels of proinflammatory mediators such as IL-6, high-sensitivity C-reactive protein (hsCRP), IL-33 and IL-1RL1 (sST2) in peritoneal dialysis patients.

## 2. Materials and Methods

### 2.1. Study Design

Thirty-seven incident peritoneal dialysis patients at the Peritoneal Dialysis Center, Department of Nephrology, Transplantology and Internal Medicine, Szczecin, Poland were enrolled in the study between 3 July 2016 and 13 January 2019. The study protocol was approved by the local Bioethics Committee (KB-0012/74/16, approval date: 27 June 2016, Szczecin, Poland). The participants were informed about the study objectives and asked to give written consent.

### 2.2. Patient Selection

All patients were recruited 4–6 weeks after they had started continuous ambulatory peritoneal dialysis treatment (CADO). Catheter implantation in each patient was performed by the same experienced surgeon using an open surgery technique. The break-in period was 2–3 weeks. All patients started renal replacement therapy with four 2 L exchanges per day using 1.36% glucose-based dialysates with a calcium concentration of 1.25 mmol/L (Baxter Healthcare (Hong Kong, China) or Fresenius Medical Care (Bad Homburg, Germany)). They presented no signs of overt infection and had not used antibiotics or anti-inflammatory drugs since the time of catheter implantation. None of the patients had experienced peritonitis since the time of catheter implantation until the day of examination.

### 2.3. Data Collection

The charts of the patients were reviewed. The following parameters were recorded: age, gender, weight (measured when dialysate was drained out), body mass index (BMI), type of nephropathy and residual renal function. The use of antihypertensive drugs was also reported. Hypertension was present in 35 patients (94%) and secondary hyperparathyroidism in 22 patients (59%). Some 12 patients had diabetes (32%); 6 of them were diagnosed with diabetes type 1 and the remaining 6 with diabetes type 2.

The causes of ESRD were as follows: diabetes (12), chronic glomerulonephritis (10), hypertension (5), autosomal dominant polycystic kidney disease (3) and chronic pyelonephritis (2). In five patients, the etiology of the disease remained unknown.

### 2.4. Procedure

On the day of the control visit at the Peritoneal Dialysis Center, a fasting blood sample was collected from each patient, 4–6 weeks after the onset of peritoneal dialysis therapy. Before the sample collection, the patients were fasting; their physical activity was as usual. Measurements of biochemical parameters and IL-6 were performed using routine laboratory techniques (Architect c8000, Abbott (Green Oaks, IL, USA)). Blood samples for cytokine measurement were centrifuged within 15 min after collection. Plasma and dialysate samples for cytokine measurement were immediately stored at −70 degrees centigrade in a freezer until further analysis.

OPG and cytokine (IL-6, IL-33 and IL-1RL1 (sST2)) concentrations were measured in plasma and in dialysate samples obtained after 4 h of a peritoneal equilibration test (PET) performed as standard during the visit.

OPG concentrations in plasma and dialysate samples were quantified with a commercial enzyme immunoassay (BioVendor, Brno, Czech Republic) according to the manufacturer’s instructions. Photometric measurement was performed at the 450 nm wavelength. The minimum detectable concentration of OPG was 0.03 pmol/L. The intra-assay coefficient of variation (CV) was <3.5% and the inter-assay CV was <5.8%.

IL-1RL1 concentration (sST2) in plasma and dialysate was measured with a commercial enzyme immunoassay (Cloud-Clone Corp., Houston, TX, USA) according to the manufacturer’s protocol. Photometric measurement was performed at a 450 nm wavelength. The minimum detectable concentration of IL-1RL1 (sST2) was 22.3 pg/mL.

IL-33 concentration in plasma and dialysate was determined with a commercial enzyme immunoassay (BioVendor, Brno, Czech Republic) according to the manufacturer’s instructions. Photometric measurement was performed at a 450 nm wavelength. The minimum detectable concentration of IL-33 was 0.2 pg/mL. 

A PET test was performed with 2000 mL of 3.86% glucose dialysate. Three dialysate samples were collected during the test: immediately after the infusion of dialysis fluid into the peritoneum was completed and after 2 and 4 h. Blood samples were obtained 2 h after dialysate installation for creatinine and glucose measurement. D/P (dialysate-to-plasma) for creatinine and D/D0 for glucose were calculated at the beginning, after 2 and 4 h of the test. Ultrafiltration was measured after 4 h of test duration.

Dialysate and urine samples were delivered by patients on the day of the study for the measurement of Kt/V.

There was no control group in the study; cytokines were measured in peritoneal effluent, specific only for peritoneal dialysis.

### 2.5. Statistical Analysis

Statistica 13 software was used for statistical analysis (StatSoft, Kraków, Poland). The Spearman’s rank correlation coefficient (Rs) was used to assess correlations between quantitative variables. Statistical significance was calculated using the Mann–Whitney U test for clinical patient data. The general linear model (GLM) was used for multivariate analysis, with prior logarithmic transformation of variables with non-normal distribution. Only independent variables which correlated significantly with the dependent variable in univariate analysis were initially included into the GLM. Subsequently, backward elimination of the independent variables was performed until only those with at least borderline significant (*p* < 0.1) beta coefficients remained in the model. The beta coefficient and *p* value were reported for each independent variable. A *p* value < 0.05 was considered statistically significant. A study with 37 patients has 80% statistical power to detect as statistically significant true associations between quantitative variables corresponding to a correlation coefficient value of ±0.45.

## 3. Results

The main characteristics of the study population with parameters of peritoneal dialysis adequacy and transport are presented in Table 1.

Mean OPG and proinflammatory mediators’ concentrations in plasma and dialysate are presented in Table 2. Plasma OPG did not correlate with dialysate OPG level (*Rs* = 0.04, *p* = 0.8).

### 3.1. Relationship between OPG and Clinical Features

Plasma OPG levels did not differ between men and women (15.01 ± 3.34 pmol/L vs. 15.74 ± 3.8 pmol/L, respectively; *p* = 0.49), but they were positively associated with age (*Rs* = 0.49, *p* = 0.002). There was no correlation between plasma OPG concentrations and BMI (*Rs* = 0.26, *p* = 0.11) or diabetes mellitus (*p* = 0.12). There was no significant association between plasma OPG concentrations and residual renal function parameters, dialysis adequacy parameters, PET test results, and calcium and phosphate metabolism parameters.

### 3.2. Relationship between OPG and Proinflammatory Mediators’ Concentrations in Plasma and Dialysate

**Figure 1:**
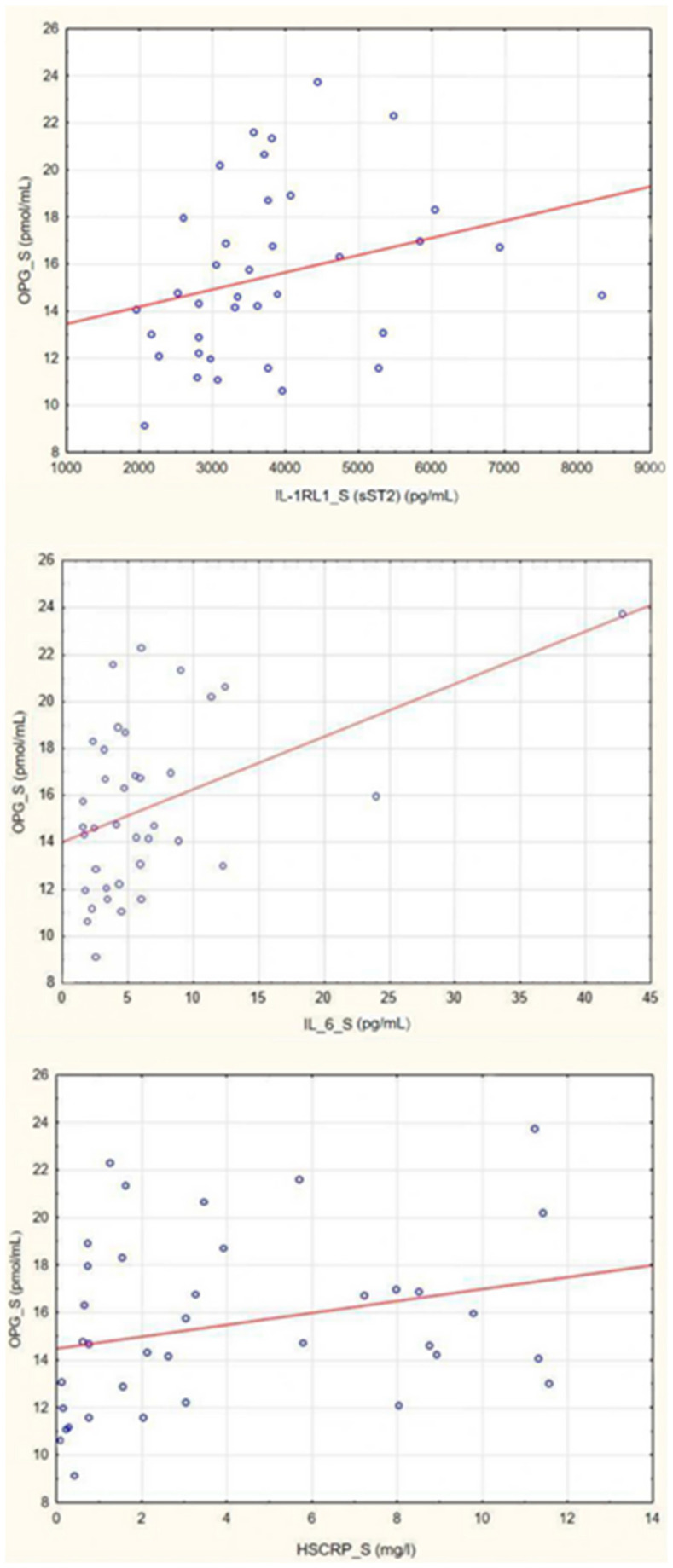
Association of plasma OPG and IL-1RL1 (sST2), plasma OPG and IL-6, and plasma OPG and hsCRP. Linear regression line was marked red.

There was a strong positive correlation between plasma OPG concentrations and plasma IL-1RL1 (sST2) (*Rs* = 0.41; *p* = 0.01), plasma IL-6 (*Rs* = 0.38; *p* = 0.01) and plasma hsCRP (*Rs* = 0.35; *p* = 0.02) (Figure 1).

**Figure 2:**
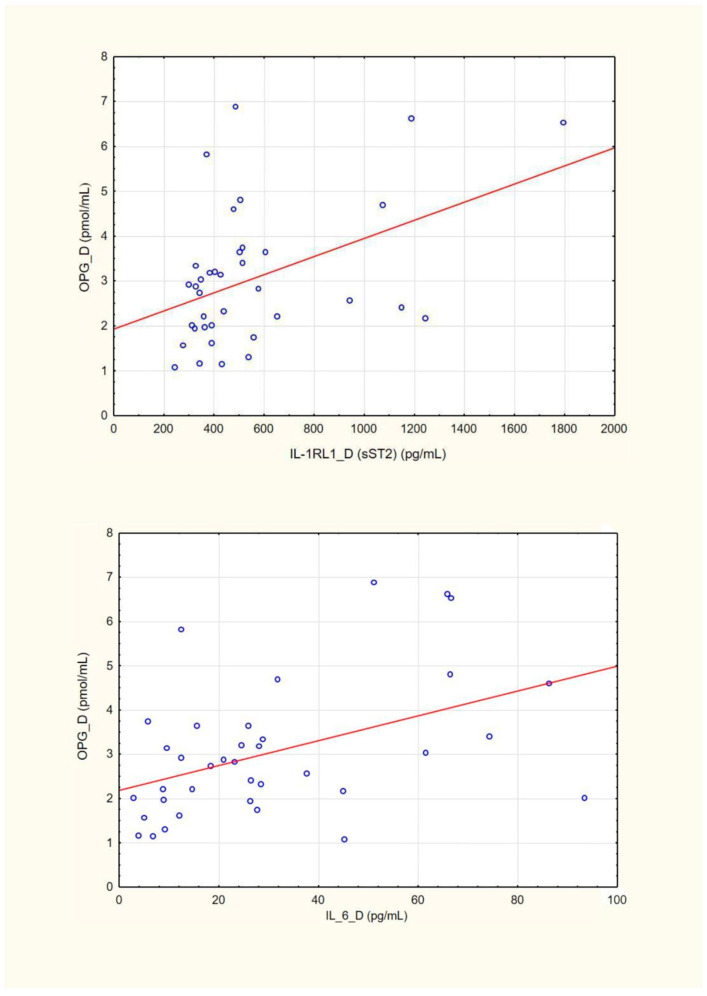
Association of dialysate OPG and IL-1RL1 (sST2) and dialysate OPG and IL-6. Linear regression line was marked red.

Dialysate OPG concentrations were positively associated with dialysate IL-1RL1 (sST2) (*Rs* = 0.37; *p* = 0.02) and dialysate IL-6 levels (*Rs* = 0.44; *p* = 0.005) (Figure 2).

### 3.3. Multivariate Analysis

Multivariate analysis was performed to establish the independent factors related to OPG plasma and dialysate concentrations. After logarithmical transformation, a higher IL-1RL1 (sST2), higher plasma hsCRP and older age were shown as independent significant determinants of higher plasma OPG concentration (Table 3).

When multivariate analysis was performed with plasma OPG as dependent variable and age and plasma IL-1RL1 (sST2) and plasma IL-6 as independent variables, logarithmically transformed higher concentrations of plasma IL-1RL1 (sST2) and IL-6 significantly predicted higher plasma OPG concentration (Table 4).

Multivariate analysis with dialysate OPG as dependent variable and logarithmically transformed dialysate IL-6 and IL-1RL1 as independent variables showed that high dialysate IL-6 was an independent significant predictor of high dialysate OPG (Table 5).

## 4. Discussion

This study gives a new insight into the associations between osteoprotegerin and proinflammatory mediators. As far as we know, this is the first time that the correlation between dialysate OPG and dialysate markers of intraperitoneal inflammation has been reported in PD patients. Our results show that both plasma and dialysate OPG levels significantly correlate with the severity of systemic and local inflammation illustrated by the concentration of IL-1RL1 (sST2), hsCRP and IL-6.

The mean plasma OPG level in the presented study group was 15.5 pmol/L, which is significantly higher compared to the healthy population. In the study of Ozkok et al., the mean OPG concentration in healthy individuals was 5.82 pmol/L, achieving a level of 9.33 pmol/L in CKD stage 4 patients and 15.11 pmol/L in those undergoing hemodialysis [20]. These results are in line with a study conducted in 2014, declaring the mean OPG concentration of 7.1 pmol/L in CKD 3–5 stage patients [21]. Krzanowski et al. investigated circulating OPG levels in 23 CKD stage 5 patients and compared them to the values achieved in 36 patients treated with hemodialysis. The mean OPG concentration in the predialysis group was 5.44 pmol/L, whereas hemodialyzed patients demonstrated values of 9.37 pmol/L [22]. Kuźniewski et al. found that the concentration of OPG in hemodialysis patients is more than twice as high as in healthy controls and amounts to 13.33 pmol/L [23]. The OPG concentration in patients on peritoneal dialysis is in line with prior research. Janda et al. declared that the mean OPG level was 9.49 pmol/L in this group and reported a strong association between circulating OPG concentration and the duration of renal replacement therapy [15]. Our findings are consistent with previous results and support the concept of OPG concentration increasing with the progression of CKD [18], with its highest levels in stage 5 [24]. Several studies revealed that plasma OPG in patients undergoing dialysis was elevated regardless of the renal replacement therapy method [15,25], with values exceeding those in predialysis CKD stage 5 patients. Moreover, no difference was found between patients performing CADO and automated peritoneal dialysis (APD) and also continuous cycling peritoneal dialysis, CCPD [16]. Interestingly, in kidney transplant recipients, OPG values decreased to the level observed in the general population, corresponding with graft function. In their work, Svensson et al. concluded that the doubling of serum creatinine or graft loss may be associated with lower renal clearance of OPG [26].

In our study, dialysate OPG was not correlated with its plasma level, achieving a mean value of 3.04 pmol/L. We believe that this work is the first to evaluate OPG in the effluent of peritoneal dialysis patients. A growing body of literature has investigated the mechanism of OPG production in vascular endothelial cells and extracellular secretion. Therefore, OPG might be detected in dialysate due to its production in the process of neovascularization of the peritoneal membrane, secondary to its chronic inflammation [14,27]. Circulatory OPG may influence its dialysate level, yet the lack of correlation between OPG concentration in plasma and dialysate suggests that it is not the main source of OPG in the effluent. Additionally, plasma OPG was not related to residual renal function parameters, indicating the possibility of its tubular secretion. In a study in 2013, Benito-Martin reported that proximal tubular cells released exosome-like vesicles containing some TNF family superproteins, including OPG. Exosomal OPG levels in urine collected from CKD patients were higher than in healthy controls [28].

We found that patients’ age was the only clinical feature positively correlated with OPG concentration in plasma. In our multivariate analysis, age proved to be the independent predictor of high OPG levels, which confirms previous findings of Avila et al. [29]. Moreover, their work showed the association of both OPG and age with cardiovascular and all-cause mortality in the population of PD patients [29]. It is assumed that a strong positive correlation between plasma OPG and age might reflect the increased intensity of chronic inflammatory processes associated with aging and comorbidities.

Plasma and dialysate concentrations of hsCRP, IL-6, IL-33 and IL-1RL1 (sST2) were analyzed to assess the relation of OPG and chronic inflammation in CKD and peritoneal dialysis per se. The levels of almost all examined proinflammatory mediators included in the study were elevated and exceeded values in healthy controls [30]. A number of studies conducted in PD patients revealed major differences between systemic and intraperitoneal inflammation. The multicenter Global Fluid Study found that, compared to plasma levels, 87% of patients had significantly higher dialysate levels of IL-6 than would be expected as a result of peritoneal diffusion. Dialysate IL-6 levels also correlated with the levels of other inflammatory cytokines measured in dialysate, i.e., IL-1beta and INF-gamma, but not with their plasma concentrations. Strong associations between various inflammatory mediators in plasma and peritoneal compartments, as well as the lack of correlation between their values measured in dialysate and in plasma, indicated the independence of both inflammatory processes. Furthermore, a higher concentration of IL-6 in dialysate than in plasma suggested its local secretion. Moreover, while dialysate IL-6 concentration was an independent predictor of peritoneal membrane function measured by creatinine D/P, plasma IL-6 concentration was negatively associated with patient survival, both in the group starting therapy, as well as in the group of prevalent PD patients [8].

We report a positive correlation between the concentration of plasma OPG and plasma hsCRP, IL-6 and IL-1RL1 (sST2) in the study group. In the multivariate analysis, these cytokines proved to be independent predictors of a higher OPG level. These results correspond to other studies showing the relationship between OPG and inflammation. It appears that plasma OPG and hsCRP values are correlated regardless of CKD presence [31]. Moreover, a strong positive association of plasma OPG and hsCRP levels was demonstrated in numerous studies in CKD stages 3–5 and also in peritoneal dialysis patients [18,22]. Nascimento et al. demonstrated significant relationships between plasma osteoprotegerin concentration and the concentration of such inflammatory parameters as hs-CRP and IL-6 in a group of patients with CKD in stages 3–5, including those undergoing peritoneal dialysis; however, there was no correlation found between plasma OPG concentration and TNF-alpha [21]. Furthermore, Koo et al. showed the correlation between OPG and hsCRP in a group of 176 patients on peritoneal dialysis, highlighting that the OPG might act as a mediator in inflammatory vascular lesions [32].

**Figure 3:**
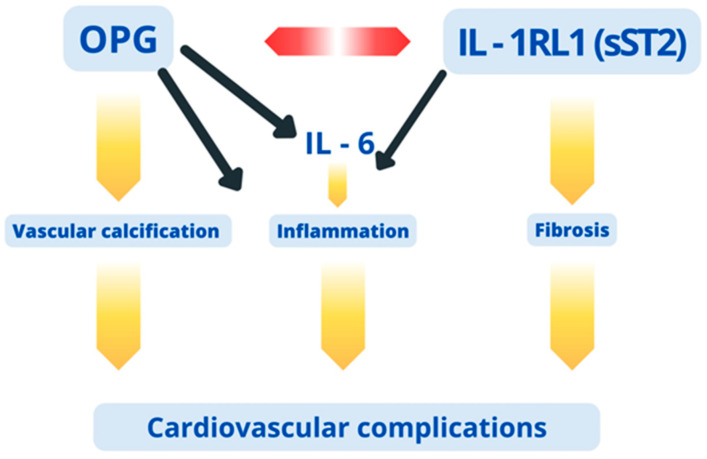
Factors and possible mechanisms contributing to the development of cardiovascular complications in CKD.

In the available literature, studies evaluating the relationship between plasma concentrations of OPG and IL-1RL1 (sST2) are lacking; this applies to both the general population and the population of CKD patients, including those on renal replacement therapy. As far as we are aware, the present study is the first to assess these parameters. IL-1RL1 (sST2), a soluble form of the receptor for IL-33, is one of the proinflammatory agents, which, affecting, e.g., the subpopulation of Th2 lymphocytes, modifies the immune response, tissue repair and remodeling processes, including fibrosis. While binding to its membrane receptor (IL 1-RACP), IL-33 activates signaling pathways such as NF-kB or MAPK and consequently stimulates myofibroblasts to express TGF-beta and other proinflammatory factors. IL-1RL1 (sST2) might be involved in inhibiting this process by preventing IL-33 from connecting with IL 1-RACP [33]. Several studies indicated that plasma IL-1RL1 (sST2) levels increased with the stage of CKD [6]. In hemodialysis patients, plasma IL-1RL1 (sST2) level is generally elevated and correlates with the risk of cardiovascular events and all-cause mortality observed in a one-year and three-year perspective [34]. The impact of IL-1RL1 (sST2) on cardiovascular mortality in patients treated with peritoneal dialysis was investigated by Choi et al. The authors suggested that a higher IL-1RL1 (sST2) level was an independent predictor of cardiovascular mortality in this group [35]. A 2021 meta-analysis of studies conducted in CKD patients confirmed those findings [36]. Our study reports a strong positive correlation between plasma OPG and IL1-1RL1 (sST2) concentration. In the multivariate analysis, higher IL1-1RL1 (sST2) levels proved to predict higher OPG levels, regardless of the model used. This may be a new element in the previously known inflammatory pathways leading to vascular calcification and fibrosis, and consequently to cardiovascular complications in patients with ESRD (Figure 3).

Moreover, in the present study, we also found relationships between the dialysate OPG and both dialysate IL-1RL1 (sST2) and IL-6. To our knowledge, this is the first study to evaluate those associations and indicate the role of OPG in local, intraperitoneal inflammation. Specifically, the correlation of dialysate OPG and IL-6 levels supports this statement, as IL-6 is the only cytokine with proven local, intraperitoneal production, affecting the peritoneal solute transport rate (PSTR) [8]. Little research has been conducted so far on the dialysate IL-1RL1 (sST2) level in peritoneal dialysis patients. Kim et al. examined IL-1RL1 (sST2) in dialysate after the first month of peritoneal dialysis and evaluated its influence on peritoneal fibrosis and consequently technique failure [37]. Higher concentrations of IL-1RL1 (sST2) in dialysate were associated with a higher incidence of peritoneal failure as a dialysis membrane. In an additional experimental study, it was found that the administration of IL-1RL1 (sST2) antibodies weakened fibrosis processes, being the result of high concentrations of glucose in the culture of human peritoneal mesothelial cells. The authors speculated on IL-1RL1 (sST2) as being a marker of ongoing local inflammatory process and peritoneal membrane impairment as a result of fibrosis. All of these findings support the hypothesis that OPG might have a pivotal role in explaining the milieu of intraperitoneal inflammation.

This study features several important limitations. First, the examined group of patients is relatively small. Second, given the cross-sectional character of the study, the cause–effect of the relation cannot be fully determined. Also, the study design did not account for potential variations in cytokines levels; thus a single measurement as such should be interpreted cautiously. A single time point of effluent OPG, IL-6, IL-1RL1 and IL-33 may not reflect changes over time. It is worth mentioning that effluent is a demanding, low-biomass specimen and there have been few published attempts to analyze its proteome. Although the presented data require verification, the obtained results encourage further research.

## Figures and Tables

**Table 1:** Patients’ characteristics.

	Mean ± SD	Median	IQR
Age (years)	50.35 ± 13.82		
BMI (kg/m^2^)	24.41 ± 3.72		
BSA (m^2^)	1.75 ± 0.16		
Hemoglobin (mmol/L)	7.11 ± 0.71		
Hematocrit (%)	34.62 ± 3.31		
Mean corpuscular volume (fL)		88.10	4.90
White blood cell count (G/L)		6.93	3.01
Urea (mg/dL)	90.19 ± 30.74		
Creatinine (mg/dL)		4.10	2.87
Uric acid (mg/dL)	5.64 ± 1.22		
Sodium (mmol/L)	139.86 ± 3.29		
Potassium (mmol/L)	4.11 ± 0.45		
Total calcium (mmol/L)	2.29 ± 0.19		
Phosphate (mmol/L)		1.34	0.34
Parathyroid hormone (pg/mL)		212.80	207.5
FGF 23 (pg/mL)	30.65 ± 2.74		
Sclerostin (ng/mL)		1.82	1.76
Total protein (g/L)	63.24 ± 6.08		
Albumin (g/L)	36.10 ± 3.86		
Alkaline phosphatase (U/L)		81.00	36.00
Total bilirubin (mg/dL)		0.40	0.22
Total cholesterol (mg/dL)		213.00	72.00
LDL cholesterol (mg/dL)		129.00	61.00
HDL cholesterol (mg/dL)	56.05 ± 14.67		
Triglycerides (mg/dL)		127.00	44.00
Glucose (mg/dL)		95.00	50.00
Residual diuresis (mL)	1848.91 ± 936.24		
Renal Kt/V		1.45	1.31
Dialysate Kt/V	1.42 ± 0.43		
Total Kt/V		2.93	1.50
UF (mL)	645.40 ± 274.83		
PET D/P creatinine	0.69 ± 0.08		

Depending on the distribution of the data, measurements were expressed either as M ± SD or Me (Q25–Q75); BMI—body mass index; BSA—body surface area; FGF 23—fibroblast growth factor 23; UF—ultrafiltration in peritoneal equilibration test; PET D/P creatinine—dialysate/plasma creatinine ratio at 4 h in peritoneal equilibration test.

**Table 2:** Plasma and dialysate proinflammatory parameters’ concentrations in the study group.

Parameter (Unit)	Mean ± SD	Median	IQR
Plasma IL-1RL1 (sST2) (pg/mL)		3583.25	1255.70
Dialysate IL-1RL1 (sST2) (pg/mL)		432.08	210.59
Plasma IL-33 (pg/mL)		73.14	14.62
Dialysate IL-33 (pg/mL)		9.09	2.18
Plasma IL-6 (pg/mL)		4.60	4.00
Dialysate IL-6 (pg/mL)		26.00	32.70
Plasma hsCRP (mg/L)		2.64	7.22
Dialysate hsCRP (mg/L)		0.12	0.06
Plasma OPG (pmol/L)	15.50 ± 3.62		
Dialysate OPG (pmol/L)		2.81	1.64

Depending on the distribution of the data, measurements were expressed either as M ± SD or Me (Q25–Q75); OPG—osteoprotegerin; IL-1RL1 (sST2)—soluble receptor for interleukin 33; IL-33—interleukin 33; IL-6—interleukin 6; hsCRP—high-sensitivity C-reactive protein.

**Table 3:** Multivariate analysis with plasma OPG as dependent variable and age, plasma IL-1RL1 (sST2) and plasma IL-6 as independent variables.

	Plasma OPG		
Independent variables	Beta (*ß*)	Beta (*ß*) SE	*p* value
age	0.36	0.13	0.01
log_plasma hsCRP	0.32	0.13	0.02
log_plasma IL-1RL1 (sST2)	0.38	0.13	0.006

OPG—osteoprotegerin; SE—standard error; log—logarithmically transformed; IL-1RL1 (sST2)—soluble receptor for interleukin 33; hsCRP—high-sensitivity C-reactive protein.

**Table 4:** Multivariate analysis with plasma OPG as dependent variable and age, plasma IL-6 and plasma IL-1RL1 (sST2) as independent variables.

	Plasma OPG		
Independent variables	Beta (*ß*)	Beta (*ß*) SE	*p* value
age	0.30	0.15	0.05
log_plasma IL-6	0.32	0.15	0.04
log_plasma IL-1RL1 (sST2)	0.35	0.13	0.01

OPG—osteoprotegerin; SE—standard error; log—logarithmically transformed; IL-6—interleukin 6; IL-1RL1 (sST2)—soluble receptor for interleukin 33.

**Table 5:** Multivariate analysis with dialysate OPG as dependent variable and dialysate IL-6 and dialysate IL-1RL1 (sST2) as independent variables.

	Dialysate OPG		
Independent variables	Beta (*ß*)	Beta (*ß*) SE	*p* value
log_dialysate IL-6	0.37	0.15	0.02
log_dialysate IL1RL1 (sST2)	0.27	0.15	0.08

OPG—osteoprotegerin; SE—standard error; log—logarithmically transformed; IL-6—interleukin 6; IL-1RL1 (sST2)—soluble receptor for interleukin 33.

## Data Availability

The data presented in this study are available on request from the corresponding author. Access to the source dataset is only permitted to employees of the Department of Nephrology, Transplantology and Internal Medicine, Pomeranian Medical University.
